# Effectiveness of the AJCC 8th edition staging system for selecting patients with T1–2N1 breast cancer for post-mastectomy radiotherapy: a joint analysis of 1986 patients from two institutions

**DOI:** 10.1186/s12885-020-07267-5

**Published:** 2020-08-24

**Authors:** Shulian Wang, Ge Wen, Yu Tang, Yong Yang, Hao Jing, Jianyang Wang, Jianghu Zhang, Xuran Zhao, Guangyi Sun, Jing Jin, Yongwen Song, Yueping Liu, Hui Fang, Yujing Zhang, Yexiong Li

**Affiliations:** 1 State Key Laboratory of Molecular Oncology and Department of Radiation Oncology, National Cancer Center/National Clinical Research Center for Cancer/Cancer Hospital, Chinese Academy of Medical Sciences (CAMS) and Peking Union Medical College (PUMC), Beijing, 100021 P. R. China; 2 Department of Radiation Oncology, Sun Yat-sen University Cancer Center, State Key Laboratory of Oncology in South China, Collaborative Innovation Center of Cancer Medicine, Guangzhou, Guangdong province 510060 P. R. China; 3 Department of Radiation Oncology, The Third Affiliated Hospital of Guangzhou Medical University, Guangzhou, 510150 Guangzhou province P. R. China

**Keywords:** Breast neoplasm, Post-mastectomy radiotherapy, One to three positive nodes, AJCC 8th edition staging system

## Abstract

**Background:**

The role of post-mastectomy radiotherapy (PMRT) in the treatment of patients with T1–2N1 breast cancer is controversial. This study’s purpose was to evaluate the risk of recurrence of T1–2N1 breast cancer and the efficacy of PMRT in low-, medium- and high-risk groups of patients.

**Methods:**

Post-mastectomy patients with T1–2N1 breast cancer were restaged according to the American Joint Committee on Cancer Staging Manual, 8th edition (AJCC 8th ed.) staging system. Recurrence scores were generated using prognostic factors identified for loco-regional recurrence and distant metastasis in patients without PMRT, and three risk groups were identified. Rates of loco-regional recurrence and distant metastasis were calculated with a competing risk model and compared using Gray’s test. Disease-free survival and overall survival were calculated using the Kaplan-Meier method and compared using the log-rank test. The Cox proportional hazards regression model was used for the multivariate analysis.

**Results:**

Data from 1986 patients (1521without PMRT; 465 with PMRT) were analyzed. Patients without PMRT were stratified into low-, intermediate- and high-risk groups by age, tumor location, AJCC 8th ed. stage, number of positive nodes and lympho-vascular invasion. The 5-year loco-regional recurrence rate and distant metastasis rates for the three risk groups were significant at 2.5, 5.4 and 16.2% (*p* <  0.001) respectively, and 4.9, 8.4 and 18.6% (*p* <  0.001) respectively. In the high-risk group, loco-regional recurrence (*p <*  0.001), and distant metastasis (*p* = 0.044) were significantly reduced, and disease free survival (*p* = 0.004), and overall survival (*p* = 0.029) were significantly improved after PMRT. In the low- and intermediate-risk groups, PMRT had no significant effect on loco-regional recurrence (*p* = 0.268), distant metastasis (*p* = 0.252), disease free survival (*p* = 0.608) or overall survival (*p* = 0.986).

**Conclusion:**

Our results showed no benefits of PMRT in the low-risk group, and thus, omitting PMRT radiotherapy in this population could be considered.

## Background

The role of post-mastectomy radiotherapy (PMRT) in the treatment of patients with breast cancer with a tumor size ≤5 cm and 1–3 positive axillary lymph nodes (T1–2N1) is controversial. The recent meta-analysis conducted by the Early Breast Cancer Trialists’ Collaborative Group showed that PMRT significantly reduced recurrence of breast cancer, including loco-regional recurrence (LRR), and breast cancer–related mortality in patients withT1–2 N1 breast cancer [1]. However, the majority of trials included in this meta-analysis were conducted 15–20 years ago, when the LRR rate for patients who did not receive PMRT was as high as 30% [2–4]. The LRR rate for T1–2N1 breast cancer is currently 10% with the use of contemporary surgical procedures and systemic therapies [5–7]. Thus, not all patients are likely to benefit sufficiently from PMRT to justify its routine use; decisions about its use or omission must be based on the latest and best evidence. The SUPREMO trial, which examined the benefits of PMRT in patients with 1–3 involved nodes may shed light on the use of PMRT in this cohort, but the final results are not yet available [8]. An accurate recurrence model for patients receiving contemporary treatment is necessary to individualize the selective use of PMRT.

The American Joint Committee on Cancer Staging Manual, 8th edition (AJCC 8th ed.) staging system provides a more accurate stratification with respect to disease-specific survival than the anatomic staging system [9], and it might be an important prognostic factor for LRR and distant metastasis (DM). According to the AJCC 8th ed. staging system, patients with intermediate stage cancers, such as T1–2N1 are the most heterogeneous group, and are classified into prognostic stages IA to IIIA [10].

This study explored the prognostic value of the AJCC 8th ed. staging system for LRR and DM by generating recurrence scores using prognostic factors to stratify patients into different risk groups. The role of PMRT was evaluated in three different risk groups to individualize the use of PMRT for patients with T1–2N1 breast cancer.

## Methods

### Patients

**Fig. 1:**
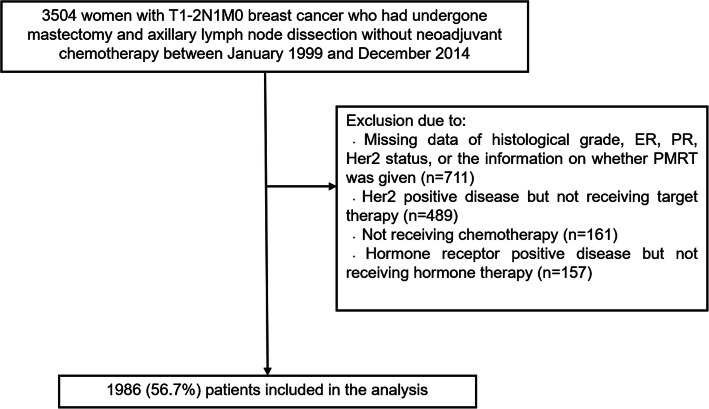
Flow chart

Patients with pathologically confirmed T1–2N1 breast cancer who underwent mastectomy and axillary dissection at two institutions in China between January 2000 and December 2014 were recruited for the study. Those who met the following criteria were included: no neoadjuvant systemic therapy, sufficient information on histological grade, estrogen receptor- (ER), progesterone receptor- (PR), or human epidermal growth factor receptor-2 (HER2) status, sufficient information on whether PMRT was provided, receipt of adjuvant chemotherapy, receipt of targeted therapy if HER2-positive and receipt of hormone therapy if ER or PR positive. A total of 1986 patients were included in the analysis (Fig. 1).

**Fig. 2:**
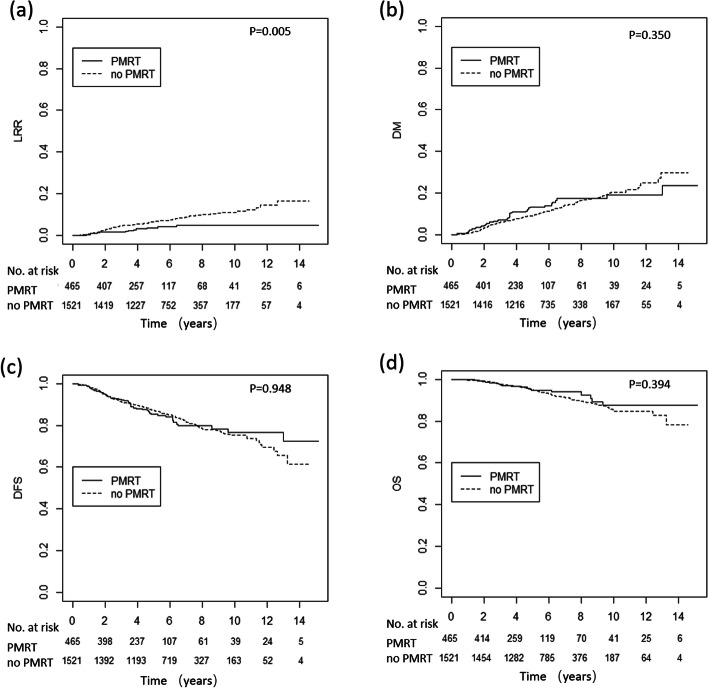
LRR, DM, DFS and OS curves for patients with and without PMRT. LRR, loco-regional recurrence; DM, distance metastasis; DFS, disease free survival; OS, overall survival; PMRT, post-mastectomy radiotherapy

### Restaging

All patients were restaged according to the newly published AJCC 8th ed. staging system for breast cancer, which incorporated biomarkers into the classical TNM system to improve discrimination. The biomarkers used included histologic grade, ER, PR and HER2-status.

### Outcomes

LRR was defined as any tumor recurrence in the ipsilateral chest wall or axillary, supraclavicular, or internal mammary nodes during follow-up regardless of their relation in time to DM. Recurrence at any other site was considered DM. Disease-free survival (DFS) was calculated from the date of mastectomy until the date of LRR, DM, death or last follow-up, whichever came first. Overall survival (OS) was calculated from the date of mastectomy to the date of death or last follow-up [11].

### Statistical analysis

The association between PMRT and patient characteristics was assessed using Pearson’s χ^2^ test. LRR and DM were calculated with a competing risk model and the differences were compared using Gray’s test. DFS and OS were calculated using the Kaplan-Meier method and the differences were compared using the log-rank test. Significant variables (*p* <  0.05) from the univariate analysis were included in multivariate analysis, which was performed using the Cox proportional hazards model. Prognostic factors for LRR and DM were identified in patients without PMRT. Points for the recurrence scores were assigned according to the Hazards ratios of the prognostic factors, which ranged from 0 points (age > 40 years, other quadrant tumor location, 1 positive node, absence of lympho-vascular invasion [LVI], stage IA), 1 point (age ≤ 40 years, inner quadrant tumor location, 2–3 positive nodes, presence of LVI, stage IB-IIA), to 2 points (stage IIB-IIIA). The points were added to determine the recurrence score, which ranged from 0 to 6 points. Patients were divided into low-risk (recurrence score = 0–1 points), intermediate-risk (recurrence score = 2 points) and high-risk (recurrence score ≥ 3 points) groups. The role of PMRT in the LRR, DM, DFS and OS of the different risk groups was evaluated. Statistical analyses were performed using cmprsk (https://cran.r-project.org/web/packages/cmprsk/) and SPSS version 24.0 (IBM Corp., Armonk, NY, USA). All *p* values were two-tailed, with a value < 0.05 considered to be significant.

## Results

### Baseline characteristics

Table 1 shows the demographic, tumor and treatment characteristics of the entire cohort, and of the subgroups with and without PMRT. The median age was 49 years (range, 23–82), the median number of axillary nodes dissected was 18 (range, 2–59) and the median number of positive nodes was 1 (range, 1–3). Invasive ductal carcinoma was diagnosed in 1953/1986 patients (98.3%). All patients received adjuvant chemotherapy, with a median of 6 cycles (range, 1–12); of them, 1377 patients (69.3%) received anthracycline and taxane-based regimens, 332 (16.7%) received a anthracycline-based regimen, 125 (6.3) received a taxane-based regimen, 55 (2.8%) received other regimens and the regimens of 49 (2.5%) were unknown. A total of 182 out of 1983 patients (9.2%) had HER2-positive disease and all of them received anti-HER2 targeted therapy with trastuzumab. All 1575/1986 patients (79.3%) who had ER and/or PR positive disease received hormone therapy, with a median duration of 48 months (range, 1–132). And 465/1986 patients (23.4%) received PMRT. Among 406 patients who had detailed RT information available, the chest wall was irradiated in 406 (100%) of them, the supra-infraclavicular nodal region was irradiated in 404 (99.5%) patients, the axilla was irradiated in 9 (2.2%) patients and the internal mammary chain was irradiated in 4 patients (1.0%). The median total dose was 50 Gy (range, 46–56) for conventional fractionation in 383 (94.3%) patients, and 40 Gy (range, 40–43.5) in 15 fractions in 23 (5.7%) patients. Compared with the no-PMRT group, the PMRT group had significantly more patients with risk factors, such as ≤40 years, AJCC 8th ed. stage IIIA, 2–3 positive nodes, < 10 nodes dissected, presence of LVI, T2, ER negative, PR negative and HER2- positive disease.

**Table 1:** Baseline characteristics of 1986 patients with breast cancer

	No. (%)	No PMRT (*n* = 1521)	PMRT (*n* = 465)	*p*
	Entire Cohort (*N* = 1986)			
Treatment era				< 0.001
1/2000–12/2009	959 (48.3)	807 (53.1)	152 (32.7)	
1/2010–12/2014	1027 (51.7)	714 (46.9)	313 (67.3)	
Age (years)				< 0.001
≤ 40	349 (17.7)	221 (14.5)	128 (27.5)	
> 40	1637 (82.3)	1300 (85.5)	337 (72.5)	
Tumor location				0.249
Inner quadrant	439 (22.1)	346 (22.7)	93 (20.0)	
Other quadrants	1526 (76.8)	1162 (76.4)	364 (78.3)	
Unknown	21 (1.1)	13 (0.9)	8 (1.7)	
Stage (AJCC 8th ed.)				< 0.001
IA	621 (31.3)	508 (33.4)	113 (24.3)	
IB- IIA	985 (49.6)	760 (50.0)	225 (48.4)	
IIB- IIIA	380 (19.1)	253 (16.6)	127 (27.3)	
No. of positive nodes				< 0.001
1	1023 (51.5)	878 (57.7)	145 (31.2)	
2–3	963 (48.5)	643 (42.3)	320 (68.8)	
No. of nodes dissected				0.002
< 10	124 (6.2)	80 (5.3)	44 (9.5)	
≥ 10	1862 (93.8)	1441 (94.7)	421 (90.5)	
Lympho-vascular invasion				< 0.001
Yes	279 (14.0)	184 (12.1)	95 (20.4)	
No	1704 (85.8)	1337 (87.9)	367 (78.9)	
Unknown	3 (0.2)	0 (0)	3 (0.7)	
Histological grade				0.004
I	76 (3.8)	69 (4.5)	7 (1.5)	
II	1309 (65.9)	1008 (66.3)	301 (64.7)	
III	601 (30.3)	444 (29.2)	157 (33.8)	
T stage				0.001
T1	953 (48.0)	762 (50.1)	191 (41.1)	
T2	1033 (52.0)	759 (49.9)	274 (58.9)	
Estrogen receptor				< 0.001
Negative	509 (25.6)	358 (23.5)	151 (32.5)	
Positive	1477 (74.4)	1163 (76.5)	314 (67.5)	
Progesterone receptor				< 0.001
Negative	542 (27.6)	369 (24.3)	173 (37.2)	
Positive	1444 (72.4)	1152 (75.7)	292 (62.8)	
HER2 status				0.002
Negative	1804 (90.8)	1410 (92.7)	394 (84.7)	
Positive	182 (9.2)	111 (7.2)	71 (15.3)	

Abbreviations: *PMRT* post-mastectomy radiotherapy, *AJCC 8th ed.* American Joint Committee on Cancer Staging Manual, 8th edition

### Treatment outcomes of the entire cohort

After a median follow-up of 68.5 months (range, 1–182 months), 147 patients died; of them, 126 (84.6%) died from breast cancer, 1 (0.7%) from treatment complications, 16 (10.7%) from other causes and 6 (4.0%) from unknown causes; 142 had LRR and 257 had DM. Compared with the no-PMRT group, the PMRT group had a lower 5-year LRR (3.6% versus 6.6%, *p* = 0.005), but a similar DM (13.3% versus 9.3%, *p* = 0.350), DFS (85.2% versus 87.3%, *p* = 0.948) and OS (94.8% versus 94.9%, *p* = 0.394) (Fig. 2). The 10-year LRR rates of the PMRT group and no-PMRT group were 5.0 and 11.2%, respectively. After adjusting for age, tumor location, T stage, number of positive nodes, number of nodes dissected, histological grade, LVI and ER-, PR- and HER2-status, the multivariate analysis showed PMRT significantly reduced LRR (hazard ratio [HR] = 0.30, 95% CI: 0.17–0.53, *p* <  0.001), and increased DFS (HR = 0.71, 95% CI: 0.53–0.96, *p* = 0.028), but had no significant influence on DM (HR = 0.83, 95% CI: 0.59–1.15, *p* = 0.258), or OS (HR = 0.64, 95% CI: 0.40–1.03, *p* = 0.066), compared with no PMRT.

**Fig. 3:**
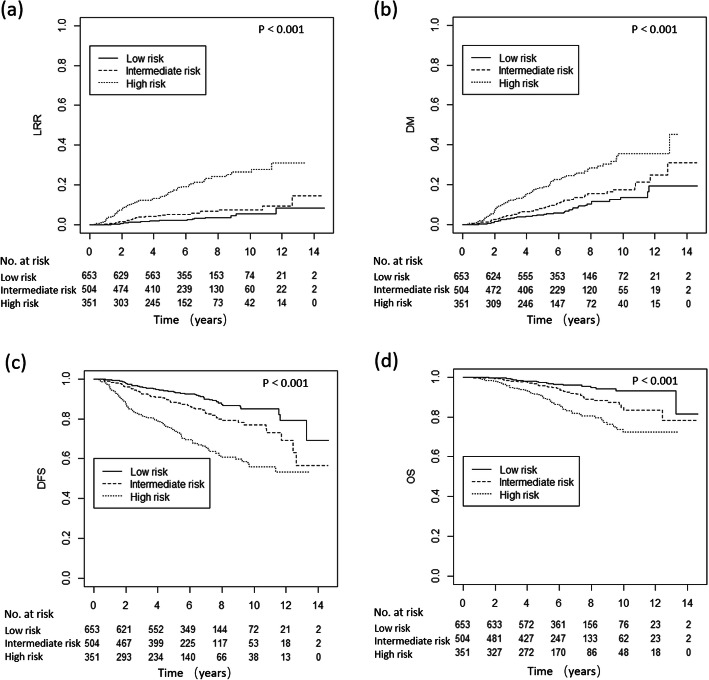
LRR, DM, DFS and OS curves for low-, intermediate- and high-risk groups without PMRT. LRR, loco-regional recurrence; DM, distant metastasis, DFS, disease free survival; OS, overall survival; PMRT, post-mastectomy radiotherapy

### Prognostic factors for LRR and DM in patients with no PMRT

The median follow-up duration for the 1521 patients who did not receive PMRT was 71 months (range, 1–175 months). Tables 2 and 3 summarize the results of the univariate and multivariate analyses of the prognostic factors for LRR and DM, respectively. Because T stage, histological grade, ER-, PR- and HER2-status were used to define AJCC 8th ed. stages, they were not included in the multivariate analysis. As a result, age ≤ 40 years, inner quadrant tumor location, 2–3 positive nodes and higher AJCC stages were independent prognostic factors for LRR. Age ≤ 40 years, inner quadrant tumor location and higher AJCC stages were independent risk factors for DM. The presence of LVI approached significance in predicting DM (*p* = 0.074).

**Table 2:** Univariate analysis of risk factors for LRR and DM in 1521 patients without PMRT

	5-year LRR % (events)	*p*	5-year DM % (events)	*p*
Treatment era		.521		0.446
1/2000–12/2009	7.2 (83)		9.9 (137)	
1/2010–12/2014	6.0 (44)		8.7 (66)	
Age (years)		.012		0.003
≤ 40	11.1 (29)		14.2 (44)	
> 40	5.9 (98)		8.5 (159)	
Tumor location		.003		0.025
Inner quadrant	10.7 (42)		11.5 (59)	
Other quadrants	5.3 (82)		8.5 (141)	
Unknown				
Stage (AJCC 8th ed.)		< .001		< 0.001
IA	2.5 (18)		4.9 (43)	
IB- IIA	6.1 (60)		8.1 (95)	
IIB- IIIA	16.7 (49)		21.9 (65)	
No. of nodes dissected		.144		0.134
< 10	10.3 (10)		13.9 (14)	
≥ 10	6.4 (117)		9.1 (189)	
No. of positive nodes		.001		0.059
1	5.7 (55)		8.7 (102)	
2–3	7.9 (72)		10.1 (101)	
Lympho-vascular invasion		.226		0.042
Yes	7.7 (17)		15.7 (28)	
No	6.5 (110)		8.6 (175)	
Histological grade		.044		0.461
I	1.6 (1)		9.3 (8)	
II	6.1 (81)		8.1 (128)	
III	8.7 (45)		11.9 (67)	
T stage		< .001		< 0.001
T1	3.7 (35)		6.8 (76)	
T2	9.7 (92)		11.9 (127)	
Estrogen receptor		< .001		< 0.001
Positive	5.0 (81)		6.7 (131)	
Negative	12.0 (46)		17.8 (72)	
Progesterone receptor		< .001		< 0.001
Positive	4.6 (74)		6.4 (126)	
Negative	13.1 (53)		18.4 (77)	
HER2		.685		0.519
Positive	7.7 (9)		8.4 (10)	
Negative	6.6 (118)		9.4 (193)	

Abbreviations: *LRR* loco-regional recurrence, *DM* distant metastasis, *PMRT* post-mastectomy radiotherapy, *AJCC 8th ed.* American Joint Committee on Cancer Staging Manual, 8th edition

**Table 3:** Multivariate analysis of risk factors for LRR and DM in 1521 patients without PMRT

	LRR	*p*	DM	*p*
	HR (95% CI)		HR (95% CI)	
Age (≤40 vs. >40)	1.75 (1.16–2.66)	.008	1.69 (1.21–2.36)	0.002
Tumor location (inner quadrant vs. non-inner quadrant)	1.92 (1.32–2.79)	.001	1.48 (1.09–2.01)	0.012
Positive lymph node (2–3 vs. 1)	1.73 (1.21–2.47)	.003		
Lympho-vascular invasion (yes vs. no)			1.44 (0.97–2.16)	0.074
Stage (AJCC 8th ed.)				
Ia	Reference		Reference	
Ib-IIa	2.10 (1.23–3.56)	.006	1.45 (1.01–2.08)	0.044
IIb-IIIa	5.95 (3.46–10.24)	< .001	3.34 (2.26–4.93)	< 0.001

Abbreviations: *LRR* loco-regional recurrence, *DM* distance metastasis, *HR* hazard ratio, *CI* confidence interval, *AJCC 8th ed.* American Joint Committee on Cancer Staging Manual, 8th edition

Recurrence scores were assigned by age, tumor location, AJCC 8th ed. stage, the number of positive nodes and LVI. There were 653 patients in the low-risk group, 504 patients in the intermediate-risk group and 351 patients in the high-risk group. A significant difference in LRR, DM, DFS and OS between the three groups was found (Fig. 3). The 5-year LRR and DM rates were 2.5, 5.4 and 16.2% (*p* <  0.001), and 4.9, 8.4 and 18.6% (*p* <  0.001), respectively. The 5-year DFS and OS rates were 93.9, 87.7 and 77.1% (*p* <  0.001), and 97.4, 95.5 and 90.3% (*p* <  0.001), respectively.

### The efficacy of PMRT in different risk groups

**Fig. 4:**
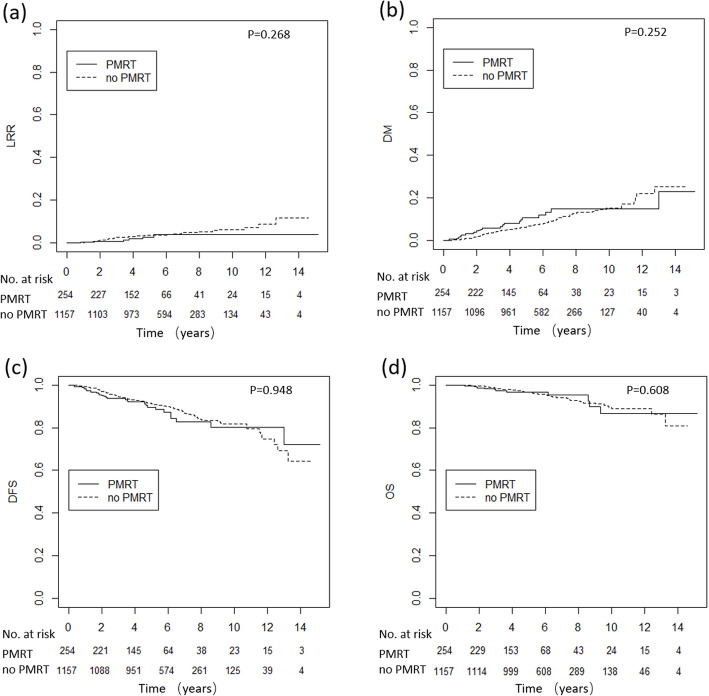
LRR, DM, DFS and OS curves for low- and intermediate-risk patients with and without PMRT. LRR, loco-regional recurrence; DM, distant metastasis; DFS, disease free survival; OS, overall survival; PMRT, post-mastectomy radiotherapy

We stratified all the patients into three risk groups based on their risk scores and compared OS, LRR and DM between the patients who did or did not receive PMRT. Among the 742 patients in the low-risk group, 89 (12.0%) received PMRT and of the 669 patients in the intermediate-risk group, 165 (24.7%) received PMRT. Among the 1411 patients in the low- and intermediate-risk groups, PMRT had no impact on LRR (*p* = 0.268), DM (*p* = 0.252), DFS (*p* = 0.608) or OS (*p* = 0.986) (Fig. 4).

**Fig. 5:**
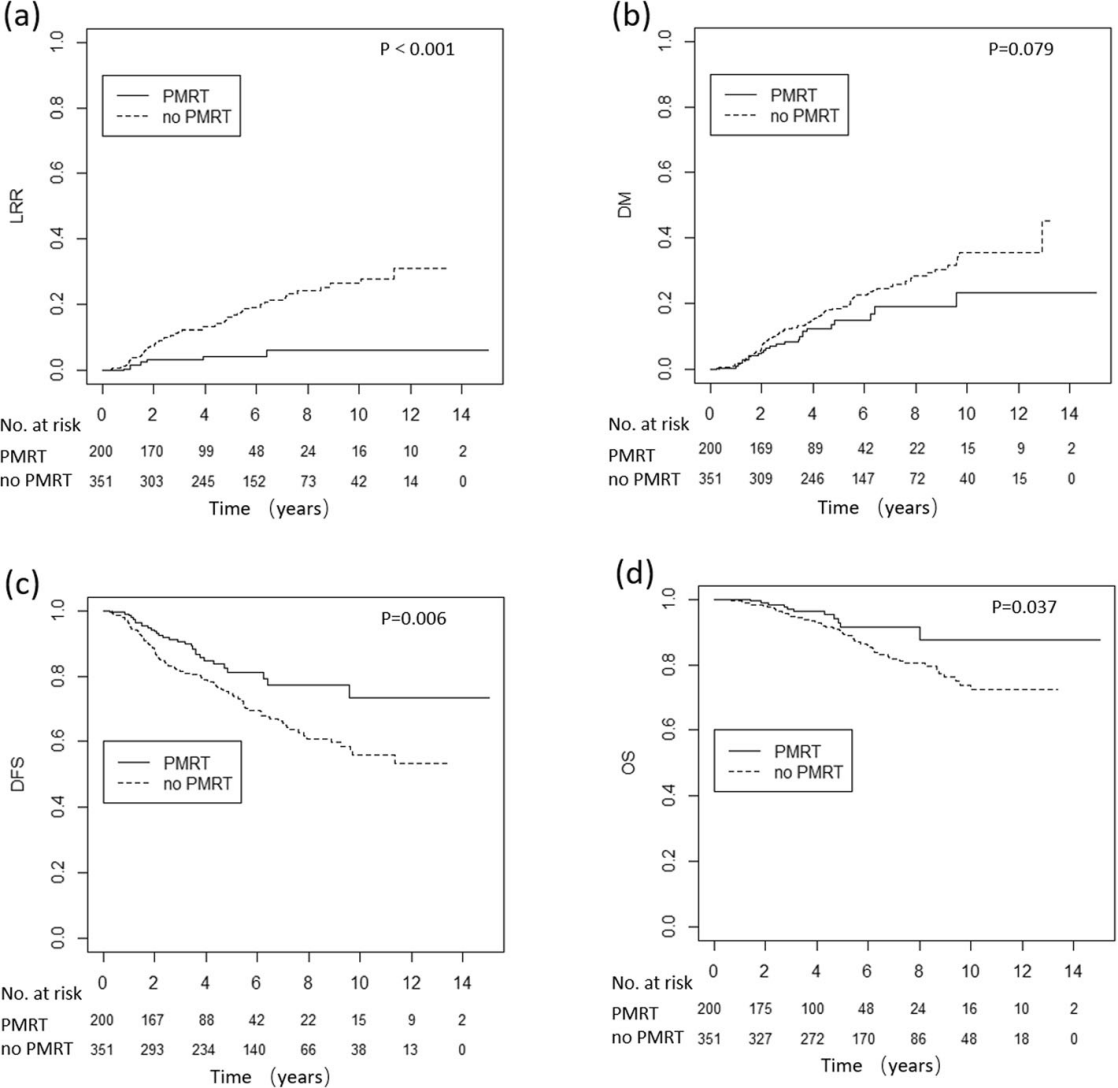
LRR, DM, DFS and OS curves for high-risk patients with and without PMRT. LRR, loco-regional recurrence; DM, distant metastasis; DFS, disease free survival; OS, overall survival; PMRT, post-mastectomy radiotherapy

Among the 551 patients in the high-risk group, 200 (36.3%) received PMRT, which significantly reduced LRR (*p* <  0.001) and improved DFS (*p* = 0.006) and OS (*p* = 0.037), but had no impact on DM (*p* = 0.079) (Fig. 5). In multivariate analysis, after adjusting for age, tumor location, number of positive nodes, LVI and AJCC 8th ed. stage, PMRT significantly reduced LRR (HR = 0.23, 95% CI: 0.11–0.49, *p* <  0.001), and DM (HR = 0.63, 95% CI: 0.40–0.99, *p* = 0.044), and it improved DFS (HR = 0.55, 95% CI: 0.36–0.83, *p* = 0.004), and OS (HR = 0.48, 95% CI: 0.25–0.93, *p* = 0.029 in the high-risk group.

## Discussion

This study is, to the best of our knowledge, the first one to establish a recurrence score for T1–2N1 breast cancer that included AJCC-8 stage as a prognostic factor, which incorporates tumor size, nodal burden and biomarkers, thereby yielding a comprehensive but simple recurrence score. We found that patients with T1–2N1 breast cancer were a heterogeneous group. They were stratified into low-, intermediate- and high-risk groups based on five prognostic factors for LRR and DM. Significant improvement was found in the outcomes of the high-risk group, which accounted for 28% of the entire cohort, but no effect was found on the outcomes of patients in the low- or intermediate-risk groups. Therefore, we recommend the selective use of PMRT for T1–2N1 breast cancer, and omitting PMRT in low-risk groups could be considered.

Recent studies have found that the risk of LRR in patients with T1–2N1 breast cancer who were not treated with PMRT was 7–15% at 10 years [7, 12]. It is likely that numerous advances in surgery, knowledge of pathology and systemic therapies have contributed to reducing the risk of LRR, such as the frequent use of sentinel node biopsy to detect small foci of metastasis, the incorporation of new chemotherapeutic regimens, targeted therapy for HER2-positive disease and endocrine therapy for ER-positive disease [13–15]. The role of PMRT should be reconsidered in current clinical practice. Data from the National Cancer Database show the proportion of patients with T1–2N1 breast cancer receiving PMRT has increased from 23.9% in 2003, to 36.4% in 2011, and that number of positive nodes and tumor size were the strongest independent predictors of PMRT use [16]. Patients with the following characteristics have been reported to have a high risk for LRR: young age (≤ 35 or < 45 or ≤ 50 years), inner-quadrant tumor location, histological grade III, ER- or PR-negative, triple-negative histology, presence of LVI, extensive intra-ductal component, extracapsular extension, high positive nodal ratio (> 15% or > 25%) and close surgical margin. However, the risk factors that were identified often varied between studies [5, 6, 12, 17–20].

We used the AJCC 8th ed. staging system to develop a simple and comprehensive scoring system for recurrence of T1–2N1 breast cancer. This staging system reflects the prognosis of patients treated using the current standard of multi-modal approaches, and is based not only on the clinical tumor burden, but also on the biomarker status of the patient [10, 21]. Therefore, this joint analysis of a large sample of patients from two institutions excluded those patients who had not received chemotherapy, HER2-positive patients who had not received targeted therapy, and ER- or PR-positive patients who had not received hormone therapy. We found that patients’ AJCC-8 stage was an independent predictor of LRR and DM among patients with T1–2N1 breast cancer. The recurrence score, which was determined by age, tumor location, AJCC 8th ed. stage, the number of positive nodes and LVI, stratified the patients into three distinct groups with significantly different prognoses for LRR, DM, DFS and OS. The 5-year rates of LRR and DM were below 5% for the low-risk group, 5–10% for the intermediate-risk group, and 15–20% for the high-risk group.

Patients with a higher risk of LRR are known to derive greater survival benefits from PMRT, provided that effective systemic therapy is delivered [22, 23]. In patients with breast cancer, PMRT could also prolong DM free survival. The NCIC (National Cancer Institute of Canada) MA.20, EORTC (European Organisation for Research and Treatment of Cancer) 22922 and Danish trials have reported a 20% relative reduction in DM with regional nodal irradiation [24–26]. Radiotherapy may eradicate loco-regional areas of disease not destroyed by systemic therapy, and these areas could be sources of eventual tumor dissemination, though active disease may not be clinically manifested at those loco-regional sites before or after systemic relapse. In this study, we identified similar prognostic factors for LRR and DM, and found that the recurrence score discriminated risk among patients with a wide range of LRR and DM rates. For those with a sufficiently low risk of LRR and DM in low- and intermediate-risk groups, the absolute reductions in LRR with the addition of PMRT was very small; thus, the routine use of PMRT is not indicated. Debate is ongoing on the recommendation to provide PMRT for patients with T1–2N1 breast cancer. In the 2019 St. Gallen guidelines, the panel recommended PMRT in cases of one to three positive nodes with a triple-negative histology, but it was divided on whether women should receive PMRT in cancers that are HER2-positive and/or ER-positive with one to three involved lymph nodes [27]. Similarly, Bazan et al. found that patients with T1 tumors and one positive LN, and patients with micro-metastases, had low event rates, such that PMRT could have been omitted [28].

Limitations of this study should be acknowledged. First, patients with worse baseline characteristics tended to receive PMRT; therefore, the no-PMRT group that we used to build the model did not represent the entire cohort of patients with T1–2N1 breast cancer. Second, the exclusion of patients who did not receive chemotherapy, endocrine therapy or targeted therapy increased the potential for selection bias; however it was helpful to link the findings of the present study to current practice. Third, we excluded patients who received neoadjuvant therapy to avoid complications in the analysis. Pathological stage cannot fully reflect the initial tumor burden after neoadjuvant therapy; the risk of LRR tended to be higher in pT1–2 N1 patients who received neoadjuvant therapy than those who did not receive neoadjuvant therapy [29]. Therefore, the considerations for PMRT should be different for pT1–2 N1 patients with and without neoadjuvant therapy. Fourth, most of the patients received PMRT to the chest wall and supra-infraclavicular nodal region, while more evidence emerged that additional internal mammary nodal irradiation further improves breast cancer outcomes [24–26], PMRT that covers extensive nodal regions might be more effective than that used in the present study. Fifth, the current analysis is based on a short follow-up of only 71 months; a more accurate recurrence pattern might have been observed with a longer follow-up period. Last, the 15-year span of patient inclusion was very long; therefore, changes in the diagnosis and treatment of breast cancer might have affected patients’ prognoses.

Nevertheless, this cohort reflects the real-world experience with a large sample size treated using current standard practices. The updated 2017 American Society of Clinical Oncology guidelines suggest that the decision to recommend PMRT to patients with T1–2N1 breast cancer should be made only after considering the specific risk factors for LRR in each patient, including the patient’s characteristics, pathologic findings and biologic characteristics. However, the panel representing the joint American Society of Clinical Oncology, American Society for Radiation Oncology and the Society of Surgical Oncology did not endorse a specific model or prescribe PMRT for a specific patient subgroup [30]. This study provides a promising recurrence model. Patients with T1–2N1 breast cancer comprise a heterogeneous group with a broad range of recurrence risk rates. We found that approximately 28% of this cohort benefitted from PMRT. As surgical techniques, pathologic evaluations and systemic therapy regimens evolve, the proportion of patients with T1–2N1 breast cancer requiring PMRT will continue to decrease. However, the relative benefits of PMRT might be greater for patients irradiated today than previously, because of better coverage of target areas achieved by modern practices in treatment planning.

## Conclusion

Our results showed no benefits of PMRT for the patients in the low-risk group, and thus, omitting PMRT in this population could be considered. These findings should be prospectively validated, as there is still a need for randomized studies.

## Data Availability

All data generated or analyzed during this study are included in this published article. The datasets used and/or analyzed during the current study are available from the corresponding author on reasonable request.

## Abbreviations

PMRT: Postmastectomy radiotherapy
AJCC: The American Joint Committee on Cancer
LRR: Locoregional recurrence
DM: Distant metastasis
ER: Estrogen receptor
PR: Progesterone receptor
HER2: Human epidermal growth factor receptor-2
DFS: Disease-free survival
OS: Overall survival
LVI: Lympho-vascular invasion
